# The Products of Pathogenic Bacteria

**Published:** 1891-03

**Authors:** 


### The Products of Pathogenic Bacteria.

During the past two years great progress has been made in the study of the action of pathogenic bacteria. This work has chiefly been carried on by Koch and Pasteur on the continent, and Drs. Sidney Martin and Hankin in England. In the *Revue de Medecine*, 1890, No. 7, Dr. Charles Bouchard publishes an article on the properties of the substances secreted by pathogenic micro-organisms. It contains the chief part of his address before the Tenth International Congress on the "Mechanism of Infection and Immunity." After an exhaustive summary of all that is known concerning the action of products of metathesis with which we are acquainted, Bouchard relates a series of thirty-one experiments which he made, partly in order to investigate the power which blood-serum possesses of destroying bacteria, and partly to ascertain how far their products confer an immunity against similar or other bacteria. Many experiments demonstrated the influence of the same products on phagocytosis. The space here is too limited to enter into the details of this interesting paper, but the general results of Bouchard's investigations are as follows: Among the substances secreted by the microbes are some which have an inhibitory action on them—that is to say, these products tend to retard the development, increase and characteristic action of the micro-organisms; other substances are favorable to their growth. These, however, only act indirectly by modifying the material upon which they grow (peptones, etc.). Such products may be favorable or unfavorable for other microbes. Some organisms produce poisonous substances upon which depends their virulence. Amongst pathogenic microbes are some which secrete snbstances that confer upon animals inoculated with them an immunity against these particular germs; this they do, not by their presence only, but by modifying the animal organism so that it forms a less favorable pabulum for the development and growth of the bacteria, and causes the leucocytes to perform the process of diapedesis more rapidly, and to assume their functions as phagocytes more energetically. If an animal be inoculated with these substances together with a pure culture of the same bacilli from which they were obtained, the disease runs a more rapid course whilst its development will be delayed or prevented if the animal be inoculated a few days before the injection is made. If bacteria which acts antagonistically towards one another be cultivated together in a test-tube, the soluble products of the "stronger" can be made to retard the development of the "weaker" organism. So that if an animal be inoculated with the products of metathesis of the "stronger" at the same time as the active principle of the "weaker," the action of the latter will be delayed and weakened. Some microbes appear to assist the action of others; these Bouchard terms "auxiliary microbes." By this means an animal may be infected with a disease which it would otherwise resist.—*Lancet.*

